# Primary care workforce composition and population, professional, and system outcomes: a retrospective cross-sectional analysis

**DOI:** 10.3399/BJGP.2021.0593

**Published:** 2022-04-05

**Authors:** Jon Gibson, Igor Francetic, Sharon Spooner, Kath Checkland, Matt Sutton

**Affiliations:** Health Organisation, Policy and Economics, School of Health Sciences, University of Manchester, Manchester.; Health Organisation, Policy and Economics, School of Health Sciences, University of Manchester, Manchester.; Health Organisation, Policy and Economics, School of Health Sciences, University of Manchester, Manchester.; Health Organisation, Policy and Economics, School of Health Sciences, University of Manchester, Manchester.; Health Organisation, Policy and Economics, School of Health Sciences, University of Manchester, Manchester.

**Keywords:** efficiency, job satisfaction, primary care, quality, skill-mix, workforce

## Abstract

**Background:**

The diversification of types of staff delivering primary care may affect professional, population, and system outcomes.

**Aim:**

To estimate associations between workforce composition and outcomes.

**Design and setting:**

Cross-sectional analysis of 6210 GP practices from a range of geographical settings across England in 2019.

**Method:**

A multivariable regression analysis was undertaken, relating numbers of staff in four groups — GPs, nurses, healthcare professionals, and health associate professionals — to patient access and satisfaction, quality of clinical care and prescribing, use of hospital services, GP working conditions (subsample of practices), and costs to the NHS. Data were obtained from the GP Patient Survey 2019, Quality and Outcomes Framework, prescribing data, the Hospital Episode Statistics database, the NHS Payments to General Practice 2019/2020, and the *Tenth National GP Worklife Survey 2019*.

**Results:**

Having additional GPs was associated with higher levels of satisfaction for the GPs themselves and for patients, whereas additional staff of other types had opposite associations with these outcomes. Having additional nurses and health associate professionals was associated with lower costs per prescription but more prescribing activity than having additional staff from the other two groups. Having more GPs was associated with higher costs per prescription and lower use of narrow-spectrum antibiotics compared with the other staff groups. Except for health associate professionals, greater staff numbers were associated with more hospital activity.

**Conclusion:**

Professional, population, and system outcomes showed a variety of associations with primary care workforce composition. Having additional nurses was associated with lower quality in some aspects, and higher costs and activity. The association between additional healthcare professionals or health associate professionals and higher costs was less than that for additional GPs, but was also linked to lower patient and GP satisfaction.

## INTRODUCTION

The supply of GPs is under strain in most countries, with workload and other pressures driving burnout and early exit from the workforce.^1^^–^^4^ One proposed solution is to broaden the workforce by introducing new types of practitioners to supplement the care provided by those who are medically qualified.^5^^,^^6^ The NHS in England has been particularly affected by these pressures^2^^–^^4^^,^^7^ and gone further than most countries in seeking to broaden the primary care workforce. A substantial investment of £746 million was pledged for 2021/2022 to employ a wide range of new types of practitioner, including pharmacists, physician associates, care coordinators, physiotherapists, and mental health support workers.^8^ However, there is limited high-quality evidence to support these developments — much of the existing research has been more narrowly focused on the mix of physicians and nurses,^9^ the performance of particular types of practitioners in their roles,^10^^–^^12^ and a narrow range of outcomes.^13^ There has been little focus on the wider impacts of employing a more diverse range of practitioners. It is not known whether such practitioners can be substitutes for GPs;^14^ likewise, their impact on the quality of care and patient satisfaction is unknown. In addition, existing evidence does not consider cost-effectiveness or the impact on care provision at practice level.^15^

Primary care in England is a good setting in which to explore the impact of skill-mix change for several reasons:

- it is delivered by a large number of small practices that have discretion over their employment, leading to considerable variation in workforce composition;
- more-detailed data on workforce composition at practice level have recently become available;^16^ and
- national policy is driving further changes in workforce and their potential impacts need to be understood.

In this study, nationwide data from a large sample of practices were used to examine whether the inputs of GPs, nurses, and other health professionals are associated with a wide range of population, professional, and system outcomes.

## METHOD

### Data

Data on different types of practitioners employed in general practices across England in September 2019 were obtained from NHS Digital, the national provider of information and data regarding health and social care in England. Information on headcounts and full-time equivalents (FTEs) were available for 35 categories of professionals providing direct patient care.

Data were obtained on outcomes from the following sources:

- the GP Patient Survey;^17^
- the Quality and Outcomes Framework (QOF);
- prescribing data;
- the Hospital Episode Statistics (HES) database;
- the NHS Payments to General Practice 2019/2020;^18^ and
- the *Tenth National GP Worklife Survey 2019* ^7^ (see Supplementary Appendix S1).

**Table:** How this fits in

The increasing number of staff from diverse healthcare backgrounds is changing the general practice workforce in England. These changes provide a new opportunity to investigate whether, and how, workforce composition may be associated with outcomes. This analysis indicated that professional, population, and system outcomes show a variety of associations with primary care workforce composition. The findings demonstrated that different types of health professionals are not substitutes for each other, and the quantity and quality of primary care services delivered will depend on who is employed to work in this setting.

All sources, except the GP Worklife Survey, provided practice-level figures for all practices in England; the GP Worklife Survey comprised a sample of individual GP responders.

Outcomes relating to the population, healthcare system, and practitioners were analysed as follows:

- population — patient experience of access (using data from the GP Patient Survey) and clinical quality (using QOF points scored and measures of prescribing quality, including the ratio of broad to narrow antibiotics prescribed and the volume of prescribing);
- healthcare system — use of hospital services, including attendance at emergency departments, emergency admissions, and hospital outpatient referral (using HES), as well as costs based on payments to practices (using NHS Payments to General Practice data), prescribing data, and costs associated with hospital activity (using national average unit costs^19^); and
- practitioners — outcomes in a subset of practices (using data from the GP Worklife Survey).

The data sources are described in more detail in Supplementary Appendix S1. Data on a range of population and practice characteristics that might be associated with workforce composition and outcomes were sourced and included as covariates. These included:

- registered population size and age structure;
- indicators of population healthcare needs;
- local area income, level of deprivation, and average wages; and
- the practice’s dispensing status, contract type, rurality, and NHS region.

Practice workforce data are self-reported by practices via the National Workforce Reporting System; if practices have not provided any data, or have provided incomplete data, NHS Digital imputes values. Only practices with 1000 registered patients that provided a complete set of workforce information were analysed.

### Analysis

Levels of staff input were measured in FTEs. The pattern of employment of new types of clinical staff in England is complicated — incentive payments are available to employ 12 different types of staff^8^ (including health coaches, clinical pharmacists, nursing assistants, and many others), and the roles undertaken by these groups vary greatly. In order to make sense of the potential impact of such a wide range of staff on patient care, the World Health Organization classification system^20^ was followed. This system not only distinguishes between those staff who are able to provide therapeutic interventions in their own right and those who carry out delegated tasks, but also classifies staff into four broad categories:

- GPs;
- nurses;
- healthcare professionals (excluding GPs and nurses); and
- health associate professionals (Table 1).

**Table 1.** WHO classification of primary care staff groups^20^

**WHO classification**	**NHS roles used in General Practice Workforce statistics**
GPs	Partners
	Salaried GPs
	Locum GPs
	Registrar GPs, Foundation 1–2, Specialty 1–4
	Retainer GPs

Nurses	Practice nurses
	Advanced and specialist nurses
	Trainee nurses

Healthcare professionals	Pharmacists
	Physiotherapists
	Physician associates
	Paramedics
	Podiatrists
	Counsellors
	Occupational and other therapists
	Other allied health professionals

Healthcare associate professionals	Dispensers
	Healthcare assistants
	Nurse associates
	Pharmacy technicians
	Psychological wellbeing practitioners
	Social prescribing link workers
	Apprentices (therapists, pharmacists, physiotherapists, and others)

*WHO = World Health Organization.*

The healthcare professionals category contains staff that make professional assessments or deliver therapeutic interventions, such as pharmacists or physician associates. The health associate professionals category contains any other staff involved in patient treatment, such as healthcare assistants, dispensers, trainees, and apprentices.

Linear regression was used to relate practice outcomes to cubic functions of the levels of each staff type.^21^^,^^22^ Interactions between GPs and each of the other three staff types were included to test for direct substitution (negative interaction coefficient) or direct complementarity (positive interaction coefficient) between staff groups. All outcome scores were standardised using z-scores to aid comparisons. All analyses were weighted by the denominator of the outcome (size of registered population for most outcomes, which is detailed in Supplementary Appendix S2).

The estimated effects of the staff variables were summarised by calculating the unit changes in the FTEs of each staff type, holding all other characteristics at their median values for continuous variables, and at their mean values for discrete variables. These indicated the marginal effects of staff changes for the average practice. Means for the outputs at key percentiles of staff input levels, conditional on the covariates, were graphed.

A similar analysis was conducted for total costs, calculated as the sum of the payments made to the practice by the NHS for providing primary care services, the costs of the prescriptions generated by the practice, and the volumes of four types of hospital activity used by the practice’s patients. To account for the skewed distribution of these costs, a generalised linear model was estimated using a log link function and a gamma distribution.^23^ This model was estimated for total costs and for six components of total costs. To check the stability of the coefficients regarding the inclusion of covariates, the model for total costs was also estimated, adding covariates sequentially.

Stata (version 15.1) was used. Huber–White robust standard errors were computed to allow for heteroscedasticity. The sensitivity of the results to allow the errors to be correlated across different outcomes, using seemingly unrelated regression equations, was also checked.^24^ Goodness-of-fit was measured using *R*^2^ and root mean square error statistics, and the joint significance of the workforce variables was estimated using *F*-tests.

## RESULTS

In September 2019, there were 6770 practices operating with 1000 registered patients, of which 6296 (93%) practices provided a complete set of workforce information. In total, 6210 (92%) practices had full data available for all covariates of this study and were included in the analysis. For the analyses using the GP Worklife Survey, the sample size varied between 1191 and 1270 responding GPs.

There was considerable variation in staffing levels across practices: the median practice employed 4.3 FTE GPs, 1.9 nurses, 0.0 healthcare professionals, and 1.0 health associate professionals (Table 2).

**Table 2.** Distribution of FTE numbers of different staff types across practices, *n* = 6210

**Staff group**	**Mean**	**SD**	**Percentile^a^**							
			**5**	**10**	**25**	**50**	**75**	**85**	**90**	**95**
GPs	5.14	3.76	1.00	1.37	2.34	4.29	7.06	8.66	9.88	11.73
Nurses	2.50	2.33	0.40	0.56	1.00	1.91	3.26	4.19	4.96	6.47
Healthcare professionals	0.30	0.77	0.00	0.00	0.00	0.00	0.16	0.80	1.00	1.71
Health associate professionals	1.57	1.94	0.00	0.00	0.48	1.00	1.97	2.77	3.23	5.15

a *Percentiles show the distribution of staff FTE in the core sample. Percentile values are the same used in the plots included in Supplementary Appendix S5. FTE = full-time equivalent. SD = standard deviation.*

Full regression results are shown in Supplementary Appendix S3 and descriptive statistics for the sample are shown in Supplementary Appendix S4. For the majority of outcomes, the staff input variables were statistically significant at *P*<0.05 — and, for the most part, at *P*<0.01. The interaction terms between the numbers of GPs and numbers of other staff are generally not statistically significant (see workforce interaction terms in Supplementary Appendix S3); this indicates a lack of substitution or complementarity for most outcomes. As exceptions, a small degree of complementarity was found between GPs and nurses in the achievement of QOF points, and between GPs and healthcare professionals in relation to outpatient attendances and time since last nurse appointment. The only substitution effects that were statistically significant were between GPs and health associate professionals for the prescribing of narrow-spectrum antibiotics and for total items prescribed.

The effects for the average practice are summarised in Table 3. The full patterns of the associations between staff levels and the outcomes are shown in Supplementary Appendix S5. The variance inflation factors were examined; these were all <10, except for the workforce and population age category variables, which would be expected to be correlated. Additional GPs were associated with shorter times since patients had a GP appointment, longer times since patients had a nurse appointment, lower proportions of work that GPs think can be delegated, and higher GP job satisfaction. Additional nurses had the opposite effects on practice functioning and working conditions. Healthcare professionals and health associate professionals had similar effects as nurses on practice functioning and working conditions; however, healthcare professionals had a much larger effect on perceived opportunities for work delegation, and health associate professionals were associated with lower GP job satisfaction.

**Figure 1.**
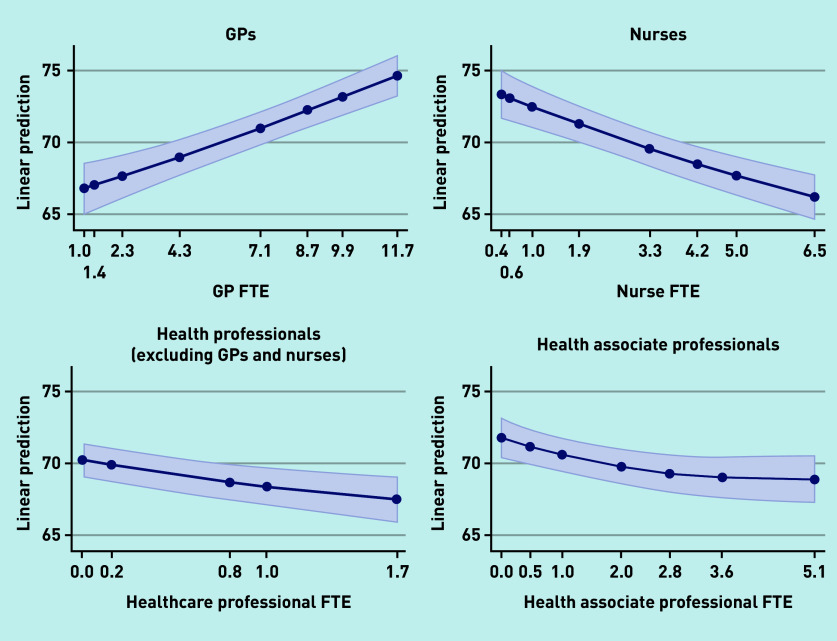
*Patient experience with making an appointment.* *FTE = full-time equivalent.*

**Figure 2.**
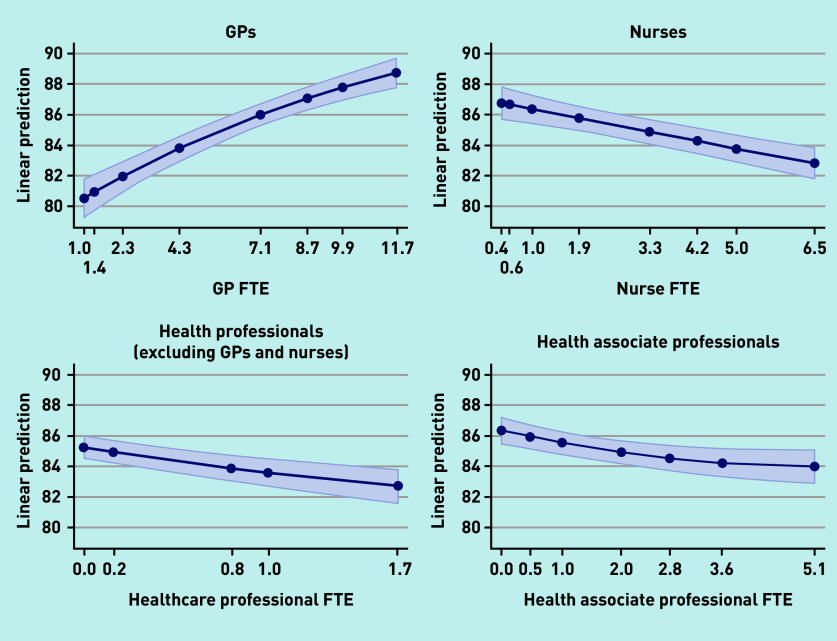
*Overall patient experience of practice.* *FTE = full-time equivalent.*

Additional staff in all four types was associated with the achievement of higher QOF points. Having additional GPs was associated with higher patient satisfaction but a greater number of staff in each of the other three groups was associated with lower patient satisfaction; Figure 1 and Figure 2 show the marginal effect of additional staff members on patient satisfaction with making an appointment and overall satisfaction with the practice, respectively.

**Table 3.** Associations between staff type and population, professional, and system outcomes^a^

**Outcome**	***n***	**Mean (SD)**	**Estimated effects of one additional FTE on z-score transformation of outcome**			

			**GPs (95% CI)**	**Nurses (95% CI)**	**Health professionals (95% CI)**	**Health associate professionals (95% CI)**
**Practice activity**						
Months since last GP appointment	6210	5.451 (0.660)	−0.043 (−0.057 to −0.028)	0.105 (0.077 to 0.134)	0.128 (0.042 to 0.215)	0.016 (−0.019 to 0.050)
Months since last nurse appointment	6210	8.139 (1.237)	0.047 (0.034 to 0.060)	−0.145 (−0.171 to −0.120)	−0.001 (−0.071 to 0.069)	−0.141 (−0.172 to −0.109)

**GP working conditions**						
Average hours worked per week by GPs	1195	41.019 (14.458)	−0.021 (−0.048 to 0.005)	0.047 (−0.014 to 0.108)	0.060 (−0.133 to 0.254)	0.010 (−0.055 to 0.074)
Percentage of GP work that could be delegated	1171	26.893 (17.649)	−0.037 (−0.064 to −0.011)	0.086 (0.026 to 0.145)	0.217 (0.016 to 0.417)	0.032 (−0.035 to 0.099)
GP job satisfaction	1270	4.428 (1.534)	0.052 (0.025 to 0.080)	0.007 (−0.052 to 0.066)	0.112 (−0.085 to 0.310)	−0.068 (−0.135 to −0.002)

**Quality of care**						
Percentage of QOF points achieved	6210	96.744 (5.596)	0.058 (0.036 to 0.079)	0.007 (−0.020 to 0.033)	0.054 (−0.039 to 0.147)	0.012 (−0.026 to 0.050)
Patient experience with making an appointment	6210	67.451 (14.349)	0.052 (0.038 to 0.066)	−0.086 (−0.113 to −0.059)	−0.157 (−0.243 to −0.071)	−0.060 (−0.092 to −0.028)
Overall patient experience of practice	6210	83.116 (9.635)	0.080 (0.066 to 0.094)	−0.068 (−0.095 to −0.042)	−0.200 (−0.294 to −0.106)	−0.066 (−0.099 to −0.034)

**Prescribing activity**						
Items prescribed per thousand patients	6210	19 021.060 (6298.436)	−0.009 (−0.020 to 0.002)	0.050 (0.033 to 0.067)	−0.062 (−0.138 to 0.013)	0.026 (0.004 to 0.047)
Cost per item prescribed in £	6210	7.478 (1.295)	0.021 (0.011 to 0.032)	−0.034 (−0.055 to −0.012)	0.030 (−0.032 to 0.092)	−0.046 (−0.074 to −0.018)
Percentage of narrow-spectrum to total antibiotics	6210	95.913 (1.538)	−0.018 (−0.030 to −0.007)	−0.015 (−0.039 to 0.009)	0.093 (0.021 to 0.164)	0.017 (−0.017 to 0.050)

**Hospital activity**						
ED attendances per thousand patients	6210	238.472 (79.267)	0.024 (0.013 to 0.035)	0.020 (−0.001 to 0.041)	0.029 (−0.033 to 0.092)	0.023 (−0.005 to 0.051)
Outpatient attendances per thousand patients	6210	1471.976 (446.682)	0.038 (0.027 to 0.050)	0.009 (−0.014 to 0.032)	0.084 (0.015 to 0.153)	−0.040 (−0.069 to −0.010)
Elective admissions per thousand patients	6210	156.607 (52.245)	0.032 (0.020 to 0.044)	0.038 (0.017 to 0.059)	0.019 (−0.051 to 0.089)	−0.022 (−0.050 to 0.005)
Emergency admissions per thousand patients	6210	90.386 (26.908)	0.041 (0.030 to 0.053)	0.055 (0.033 to 0.076)	0.075 (0.012 to 0.137)	−0.029 (−0.057 to −0.002)

a *Outcome mean and regressions are weighted by the outcome denominator (see Supplementary Appendix S2 for details). Full results from OLS regression model used to derive marginal effects are included in Supplementary Appendix S3. Margin plots showing the effects graphically for a range of staffing percentiles are included in Supplementary Appendix S5. The results of sensitivity checks are available in Supplementary Appendix S6. Full diagnostic indicators for all regression models are in Supplementary Appendix S7. ED = emergency department. FTE = full-time equivalent. OLS = Ordinary Least Squares. QOF = Quality and Outcomes Framework. SD = standard deviation.*

Additional nurses and health associate professionals were associated with more prescribing activity but lower average cost per prescription. Additional GPs increased costs per prescription and lowered the use of narrow-spectrum antibiotics, and additional healthcare professionals increased the use of narrow-spectrum antibiotics. Additional staff in primary care, with the exception of health associate professionals, was associated with more hospital activity of all types. Additional healthcare professionals had the largest impact on hospital activity, particularly on outpatient attendances and emergency admissions.

The analysis of overall practice costs (Table 4) showed that an increase of one FTE nurse resulted in the largest increase in costs (£512 000 per practice per year, 95% confidence interval [CI] = £446 000 to £579 000), followed by GPs (£362 000, 95% CI = £326 000 to £398 000) and health associate professionals (£256 000, 95% CI = £195 000 to £318 000). The effect of healthcare professionals on costs was the smallest and not statistically different from zero (£182 000, 95% CI = −£33 000 to £396 000). The effects of different staff groups were similar in terms of increasing or decreasing costs, except that healthcare professionals did not increase prescribing costs, and healthcare professionals and health associate professionals did not increase hospital activity as much as GPs and nurses.

**Table 4.** Associations between practice workforce and costs of practice to the NHS^a^

**Annual practice costs**	**Mean cost, £’000 (SD)**	**Estimated effects of one additional FTE on costs**			
		**GPs, £’000 (95% CI)**	**Nurses, £’000 (95% CI)**	**Health professionals, £’000 (95% CI)**	**Health associate professionals, £’000 (95% CI)**
Total	7950 (4808)	362 (326 to 398)	512 (446 to 579)	182 (−33 to 396)	256 (195 to 318)
NHS payments to practice	1265 (805)	49 (44 to 55)	86 (75 to 96)	41 (5 to 77)	72 (61 to 84)
Prescriptions	1261 (850)	50 (44 to 56)	78 (67 to 89)	−8 (−58 to 42)	41 (31 to 50)
ED attendances	361 (232)	15 (13 to 17)	20 (16 to 24)	9 (−2 to 19)	13 (10 to 18)
Emergency admissions	1390 (893)	71 (64 to 78)	100 (85 to 114)	46 (4 to 88)	41 (27 to 54)
Elective admissions	1927 (1263)	93 (82 to 105)	128 (108 to 148)	37 (−22 to 95)	45 (25 to 66)
Outpatient attendances	1746 (1122)	85 (76 to 94)	97 (79 to 115)	60 (10 to 111)	36 (18 to 53)

a *Full results for generalised linear regression models used to derive the marginal effects of different staff groups are included in Supplementary Appendix S3. Margin plots showing the effects graphically for a range of staffing percentiles are included in Supplementary Appendix S5. The results of sensitivity checks are shown in Supplementary Appendix S6. Full diagnostic indicators for all regression models are in Supplementary Appendix S7. ED = emergency department. FTE = full-time equivalent. SD = standard deviation.*

The seemingly unrelated regression models and other sensitivity checks confirmed the main results (see Supplementary Appendix S6).

## DISCUSSION

### Summary

The composition of the workforce in primary care practices was statistically significantly associated with most indicators of professional, population, and system outcomes, but the nature of these associations varied across outcomes. The number of GPs was associated positively with both GP job satisfaction and patient satisfaction, whereas the numbers of nurses, healthcare professionals, and health associate professionals related to these outcomes in the opposite direction. Additional nurses and health associate professionals was associated with greater prescribing activity, but a lower average cost of prescribing. In terms of narrow-spectrum antibiotics, additional GPs was associated with lower use, whereas additional healthcare professionals was associated with higher use. With the exception of health associate professionals, additional staff in primary care was associated with greater levels of hospital activity per head of population. Additional staff increased the costs of the practice to the NHS, with the largest effect being for nurses and the smallest effect being for healthcare professionals.

Although the numbers of staff in each group were associated with outcomes, the effects were largely independent of the numbers of staff in the other groups. There were few instances where there was evidence of statistically significant substitution or complementarity between GPs and the other three staff groups.

### Strengths and limitations

Nationwide data from a large number of primary care practices were used and, because practices are generally small organisations, both in the study and in general, with substantial discretion over how they organise services, there was a great degree of variation in workforce composition to analyse. Data were obtained from several sources, and professional and patient satisfaction, as well as indicators of healthcare activity and quality, were considered. In addition, how workforce composition was associated with costs was explored. Adjustments were made for several population and practice characteristics that were associated with workforce composition and outcomes.

In analysing the relationship between workforce composition and outcomes, a flexible regression model was used. This cubic function has been shown to be an acceptable alternative to the most flexible translog specification used in economic studies of production and less susceptible to multicollinearity in applied work because it requires fewer interaction terms.^20^^,^^21^ Although the cubic production function, like many flexible functions, resulted in correlated variables, it was their combined effect that was of interest, rather than the coefficients for individual measures, which might be estimated imprecisely because of multicollinearity.

The authors relied on workforce data collected by the NHS and worked closely with the data provider to identify practices whose values had been imputed because of known problems with missing or incomplete data; this affected 7% of practices and these were excluded from the analysis. A further 1% of practices were excluded because of missing practice characteristic data.

National data on primary care activity (for example, numbers of patient consultations) were not available at practice level. Instead, indicators from the GP Patient Survey on how many months ago responders had last seen a GP and last seen a nurse were used; these showed the expected associations with numbers of GPs and nurses, but better-quality data might have shown stronger relationships.

Not all aspects of costs that practices create for the NHS were included. However, the six cost components that were all included showed similar patterns, which suggests the results are not driven by one aspect of cost. It has not been possible to comment on health and social care expenditure more generally, although it may be, for example, that increased expenditure in primary care results in lower costs in social care.

Although several population and practice characteristics that may, otherwise, have confounded the relationship between workforce composition and outcomes were included, this was a cross-sectional study and may be prone to bias from unmeasured confounding.

### Comparison with existing literature

It has been shown that GP practices that are well organised tend to deliver high-quality care,^25^ and tasks that can be guided by clinical protocols can be successfully delegated to nurses (for example, chronic conditions management).^9^^,^^26^^,^^27^ This is consistent with the finding presented here of a small degree of complementarity between GPs and nurses in the achievement of QOF points. However, it remains unclear whether the overlap in roles performed by nurses described by Lukewich *et al* also extends to other staff groups and whether this enhances care.^28^

The finding presented here that workforce composition is associated with mixed effects on the use of hospital services is consistent with research, highlighting the complexity of this area.^29^^,^^30^ In addition, the fact that both positive and negative outcomes were associated with the healthcare professional group is consistent with prior evidence that highlighted the challenge of managing interprofessional interactions and negotiating role boundaries and regulations.^31^ The results presented here also confirm the positive effects of health professionals, which includes pharmacists as the largest subgroup, on prescribing practices and patient safety;^32^ however, broader integration of pharmacists in practice seems to be still lacking.^33^^,^^34^

### Implications for research and practice

Current health policy in England continues to drive changes in workforce composition with the employment of ever-increasing numbers of workers through the Additional Roles Reimbursement Scheme (ARRS). However, the present analysis indicates that deployment of newer types of practitioners cannot be assumed to provide a straightforward, complete, or cost-neutral solution to the GP workforce and workload crisis.

These findings highlight the importance of professional workers in the production of health care. They demonstrate that different types of health professionals are not simple substitutes for one another, and that the quantity and quality of services generated in primary care will depend on who is employed to work in this setting.

GP practices now face a more complex set of challenges when selecting from different types of health professionals to, for example, maximise the gains of skill-mix for their patient population; adjust workloads to improve GP recruitment and retention; or maintain practice income by achievement of incentivised quality targets. It follows that practice managers may require additional training as they manage increasingly diverse teams.

Further research is also needed to analyse changes in outcomes (and related overall costs) over time because of continuing changes in workforce composition. It will be particularly important to track impacts on other measures of overall health service use that may become increasingly evident with the passage of time.

Very little evidence of direct interactions between the effects of different types of workers on outcomes at the organisation level was found. This may indicate that different staff groups are not coordinating their work and are working quite independently. As such, there may be gains to be made from encouraging more effective team working.

The effects of workforce composition were examined in relation to the work experiences of GPs only because the available survey data are collected only from this staff group. Future work should consider the effects of workforce composition on the working lives of other primary care practitioners.

Statistically significant associations between cross-sectional variations in workforce composition across primary care practices and a range of outcomes have been shown. As workforce composition continues to diversify over time, it will be important to examine how this affects outcomes and costs.

## Data

The analysis in this article is based on publicly available data, apart from Hospital Episode Statistics, which is provided by NHS Digital for a fee.
